# A randomized phase I study comparing the pharmacokinetics of a bevacizumab (HD204) biosimilar to European Union- and United States of America-sourced bevacizumab

**DOI:** 10.1371/journal.pone.0248222

**Published:** 2021-09-23

**Authors:** Martin Demarchi, Pierre Coliat, Philippe Barthelemy, Roland Schott, Meher BenAbdelghani, Michael Kim, Jocelyn Chung Shii Hii, Peggy Feyaerts, Felicia Rui Xia Ang, Marie Paule Derde, Filip Deforce, Thierry Petit, Chris Schwabe, Chris Wynne, Lisa Soyeon Park, Xavier Pivot

**Affiliations:** 1 Paul Strauss Center, Strasbourg, France; 2 Institut Cancérologie de Strasbourg (ICANS), Strasbourg, France; 3 Prestige BioPharma Ltd, Singapore, Singapore; 4 DICE Cro bvba, Brussels, Belgium; 5 Auckland Clinical Studies, Auckland, New Zealand; 6 Christchurch Clinical Studies Trust Ltd, Christchurch, New Zealand; University of Sydney, AUSTRALIA

## Abstract

**Purpose:**

This first-in-human study was designed to evaluate the pharmacokinetic (PK) equivalence between HD204 and the European Union (EU)-sourced bevacizumab, between HD204 and the United States of America (US)-sourced bevacizumab, and between EU-sourced and US-sourced bevacizumab (NCT 03390673).

**Methods:**

In this randomized, double-blind, 3-way parallel group, single-dose comparative PK study, healthy male subjects were randomized to receive a single 1 mg/kg intravenous dose of HD204, EU-sourced bevacizumab or US-sourced bevacizumab. PK parameters were calculated using non-compartmental methods. PK equivalence was determined using the pre-defined equivalence margin of 0.8–1.25 in terms of AUC_(0-∞)_ for the pairwise comparisons.

**Findings:**

Baseline demographics for the 119 randomized subjects were similar across the three groups. The 90% CIs for the ratio of the geometric means of HD204 to US-sourced bevacizumab, HD204 to EU-sourced bevacizumab, and EU-sourced to US-sourced bevacizumab were all within the interval of 80% to 125% for AUC_0-inf_, thus demonstrating equivalency in the PK properties for all three treatment groups. Similarly, the ratio of the geometric means for AUC_0-last_ and C_max_ were all within the 80% and 125% margins, supporting the robustness of the primary findings. All other PK parameters, including the half-life (t1⁄2) clearance (CL), volume of distribution (Vd) and time of maximum concentration (t_max_), were comparable. There was no difference between the 3 treatment arms in terms of vital signs, laboratory tests and adverse events. None of the subjects treated with HD204 had positive ADA results.

**Implications:**

HD204 demonstrates equivalent pharmacokinetic profiles compared to those of both US-sourced and EU-sourced bevacizumab. (NCT 03390673).

## Introduction

Angiogenesis is an important physiological process during which new blood vessels are formed from pre-existing vessels. Under normal physiological conditions, angiogenesis is tightly regulated, but pathological angiogenesis, a hallmark of oncogenesis, plays a crucial role in providing oxygen and nutrients to tumour cells to facilitate tumour progression. Many pro-angiogenic factors and their cognate receptors have been found to be upregulated in various cancers and are thus potential therapeutic targets. Among them, vascular endothelial growth factor (VEGF) is regarded as a key regulator of tumour angiogenesis. Bevacizumab is a monoclonal antibody recombinant humanized immunoglobulin G with a kappa light chain (IgG1ĸ) that binds to VEGF and inhibits recognition by its receptors, Flt-1 (VEGFR-1) and KDR (VEGFR-2), present on the surface of endothelial cells [1]. Neutralizing the biological activity of VEGF can either induce regression of the vascularization of tumours, normalize the remaining tumour vasculature or inhibit the formation of new tumour vasculature, thereby inhibiting tumour growth. In 2004, the first approval of this drug for routine clinical use was obtained in first-line metastatic colorectal cancer and has been followed by demonstration of efficacy in numerous cancers and clinical situations. Currently, bevacizumab is approved and routinely given for the treatment of metastatic colorectal cancer, non-small-cell lung cancer (NSCLC), renal cell cancer, ovarian cancer and metastatic breast cancer. Bevacizumab is also approved in the United States for the treatment of recurrent glioblastoma multiform. In addition, efficacy has been established in advanced cervix carcinoma as well as in advanced endometrium cancer [1,2].

Therapeutic antibodies like bevacizumab are expensive and contribute to the rising cost of cancer care which induce important financial pressure on healthcare systems. In numerous countries, costs may prohibit access to these biotherapies and definitively not all patients receive bevacizumab as indicated, with detrimental consequences. The emergence of bevacizumab biosimilars with lower cost, might contain the rising healthcare expenditure and improve access to biotherapies. Recently, patent expirations for reference bevacizumab have occurred in 2020 for Europe and 2019 for USA allowing the routine use for several biosimilar versions of bevacizumab. Biotherapies are structurally complex molecules produced in living systems using complex manufacturing processes and cannot be identically replicated. A narrow path defining the acceptable similarity between the biosimilar candidate and the referent medical product (RMP) is specified by the regulatory agencies with stringent recommendations for the overall comparability exercice [3–5]. The goal is to warrant comparable safety and efficacy for the biosimilar in comparison with the RMP. These guidelines recommend a stepwise approach in developing a biosimilar starting with extensive *in vitro* physicochemical and biological characterization before initiating pre-clinical comparison of biological activity, efficacy, safety and pharmacokinetic (PK) of the biosimilar. Supported by evidence obtained from the non-clinical studies, the ultimate steps are comparative clinical studies aimed to demonstrate the PK equivalence in human and later the clinical equivalence in terms of efficacy and safety in a sensitive clinical indication of the RMP.

HD204 is a biosimilar candidate of bevacizumab (Avastin®). Extensive comparability exercises have been performed to demonstrate similar physicochemical, functional and biological characteristics between HD204 and EU-sourced or US-sourced bevacizumab (data in press). A pharmacokinetic/pharmacodynamic study in cynomolgus monkeys established that there was no significant difference between HD204 and EU-sourced as well as US-sourced bevacizumab (data in press). The present manuscript provides the results of the first-in-human study designed to evaluate the PK equivalence between HD204 and EU-sourced and US-sourced bevacizumab [3,4].

## Methods

### Study design

**Fig 1:**
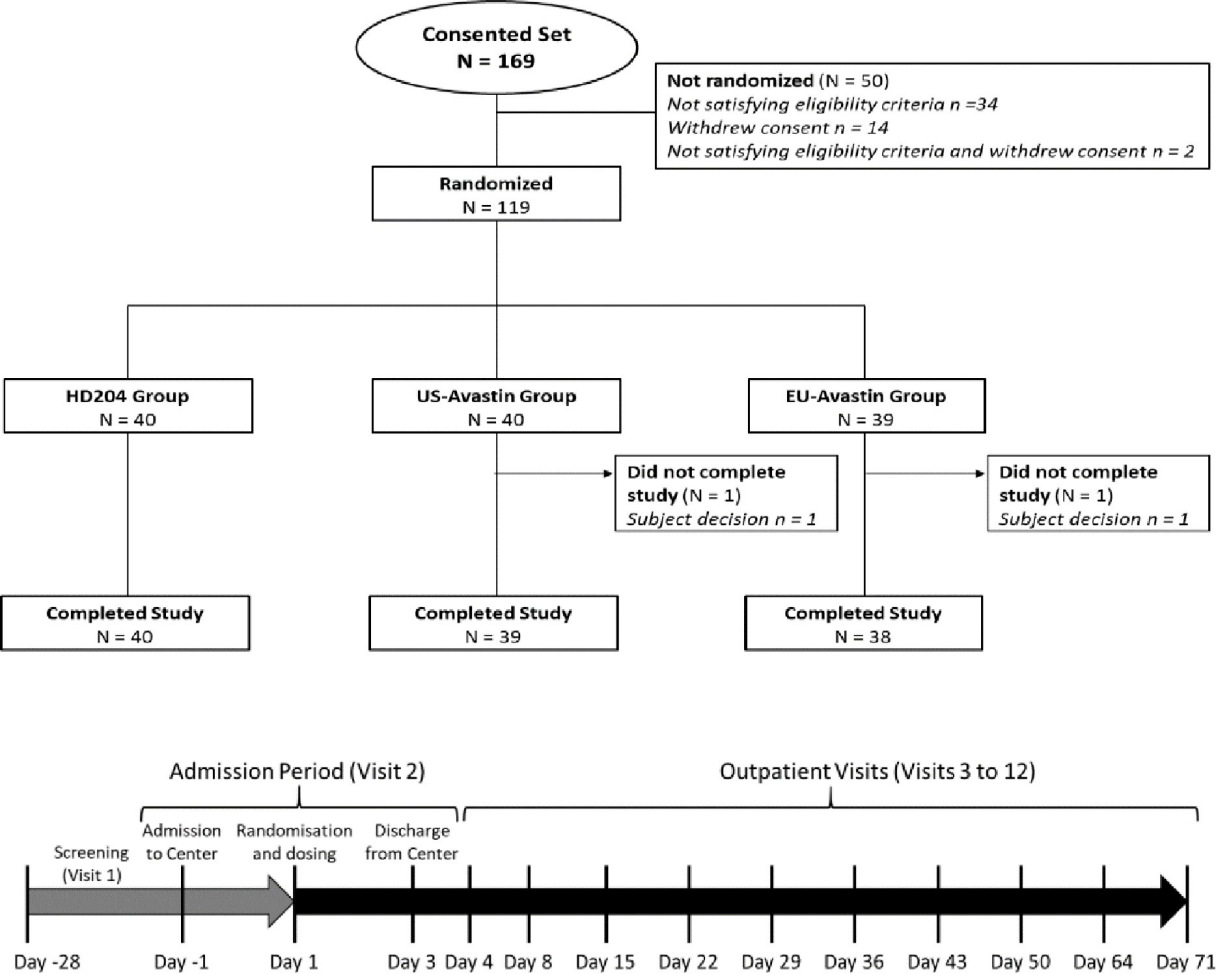
Study design. N = number of subjects. Pharmacokinetic schedule: Prior to infusion of study drug, 45 minutes after the start of study drug infusion, at the end of infusion, at 3h, 6h, and 12h after the end of infusion, at 24h (Day 2), 48h (Day 3), 72h (Day 4), 168h (Day 8), 336h (Day 15), 504h (Day 22), 672h (Day 29), 840h (Day 36), 1008h (Day 43), 1176h (Day 50), 1512h (Day 64), 1848h (Day 78) and 1680h (Day 71).

This study was a double-blind, three-arm, parallel group, single-dose study in healthy male subjects (Fig 1).

The primary objective of the study was to establish pairwise PK similarity in terms of area under the concentration-time curve in serum from zero and extrapolated to infinite time (AUC_0-inf_) between HD204 (Prestige Biopharma Pte. Ltd bevacizumab), EU-sourced bevacizumab (Avastin®, F. Hoffman-La Roche, Basel, Switzerland) and US-sourced bevacizumab (Avastin®, Genentech Inc, San Francisco, CA, USA) after a single intravenous (IV) infusion of 1 mg/kg over a 90-minute IV infusion on Day 1. The secondary objectives were to compare all PK characteristics, including the C_max_ and AUC_0-tlast_, as well as the safety, tolerability, and immunogenicity of HD204, EU-sourced, and US-sourced bevacizumab. This study was conducted in compliance with Good Clinical Practice and the Declaration of Helsinki. The study protocol and its amendments were approved by the Independent Ethics Committee of New Zealand, and all of the participants provided written informed consent prior to any study-related procedures (NCT 03390673).

For inclusion in the study, subjects had to be between the ages of 18 and 50 years and body weight between 60.3 and 99.9 kg (body mass index between 18.9 and 30.0 kg/m^2^). All subjects had to have normal screening results for vital signs, physical examination, laboratory tests including serology, haematology, chemistry, urinalysis, and urine drug screening and to display systolic blood pressure ≥ 90 and ≤ 140 mmHg, diastolic blood pressure ≥ 50 and ≤ 90 mmHg and heart rate ≥ 40 and ≤ 90 bpm at screening and admission on Day 1. Subjects who had a history of cardiac disease, cancer, or any clinically significant disease; the presence of proteinuria; or known personal or history of venous thromboembolic events or idiopathic venous thromboembolic events in a first-degree relative were excluded. Subjects with previous exposure to any monoclonal antibody or fusion protein were also excluded from the study. Included subjects were assigned by a block randomization method between HD204, EU-sourced and US-sourced bevacizumab arm.

Based on historical data, an inter-subject variability of 25% and a proportion of 20% of non-PK evaluable subjects have been assumed for sample size determination. Assuming an inter-subject geometric coefficient of variation of 25% and a geometric mean ratio of 1.05, 40 evaluable subjects per arm are required for the 2-sided 90% confidence interval (CI) of the geometric mean ratio to be completely contained within 80% to 125% with at least 90% power for each pairwise comparison. To allow for a proportion of 20% non-PK evaluable subjects, 150 subjects (50 subjects per arm) were initially planned to be enrolled.

Due to the uncertainty in the variability of the primary PK variable, a blinded sample size re-evaluation (BSSR) was performed after 120 evaluable subjects (approximately 40 per arm) completed the study. If the BSSR showed that the total variability was at most equal to the planned variability of 25% considered for the sample size calculation, the study was to be stopped. Otherwise, additional subjects were recruited.

### Pharmacokinetic evaluations

Blood samples were collected from subjects to determine the serum concentration of bevacizumab prior to infusion and then at 0.75, 1.5, 3, 6, 12, 24, 48, 72, 168, 336, 504, 672, 840, 1008, 1176, 1512, 1848 and 2256 hours after the start of infusion. Derivation of the PK parameters for bevacizumab in serum was performed by the clinical pharmacokinetist at IQVIA, Overland Park, Kansas, United States.

AUC_0-inf_ was estimated by linear up/log down trapezoidal summation and extrapolated to infinity by addition of the last quantifiable concentration (C_last_) divided by the terminal rate constant (z), AUC_0-last_ + C_last_/(_z._ The area under the concentration-time curve from zero to the last quantifiable concentration (AUC_0-last_) was calculated using the linear trapezoidal rule, using actual elapsed time values. Taking into account that the limit of quantification was 350 ug/L, the maximal concentration (Cmax) was obtained directly from the observations. The half-life (t1⁄2) was calculated as ln(2)/z. Clearance (CL) was calculated as Dose/AUC_(0-∞)_, and the volume of distribution (Vd) was calculated as Dose/z* AUC_(0-∞)._ The time of maximum concentration (t_max_) was obtained directly from the observed concentration versus time data.

### Statistical analysis

The primary statistical null hypothesis is that AUC_0-inf_ is equivalent between HD204 and the reference products within 80% to 125% limits, against the alternative that HD204 is not equivalent to the reference products. The primary PK parameter, AUC_0-inf_, is compared using an analysis of variance (ANOVA) model with treatment as a fixed effect. The data are natural log transformed prior to the analysis following the guidance from the regulatory authorities. The normality of their distributions were tested by Shapiro-Wilk, Kolmogorov-Smirnov, Cramer-von Mises and Anderson-Darling tests. Transformed back to the original scale, the geometric mean together with the 2-sided 95% CI for each treatment is estimated and presented. Additionally, ratios of geometric means together with CIs (2-sided 90%) for treatment comparisons (HD204 versus EU-sourced, HD204 versus US-sourced, EU-sourced versus US-sourced bevacizumab) are presented. As foreseen in the protocol and the statistical plan, no correction for multiplicity was applied in the analysis. The analysis was repeated for the secondary PK parameters C_max_ and AUC_0-last._

### Safety evaluations

All adverse events reported during the study were coded according to the Medical Dictionary for Regulatory Activities (Version 16.1). Severity was graded according to the National Cancer Institute Common Toxicity Criteria for AE version 4.0 (NCI-CTCAE v4.0).

#### Immunogenicity evaluations

Blood samples for analysis of the incidence and titre of antibodies to bevacizumab and the incidence of any NAbs in serum were collected.

Subjects who were confirmed positive for anti-bevacizumab antibodies at the follow-up visit (Day 71) or at an early termination visit (subjects who discontinued the study following drug administration) were to be followed up for up to 12 months or until 2 consecutive samples were confirmed negative for anti-bevacizumab antibodies, whichever occurred earlier. These subjects were to attend the study centre at 6, 9, and 12 months after study completion post-dose for blood sampling to confirm their immunoglobulin status. According to the protocol, both the incidence and titre of antibodies to bevacizumab were to be reported. The presence of ADAs was reported as positive or negative without the corresponding titres. Also according to the protocol, the incidence of any NAbs in serum from subjects with positive ADAs was to be reported.

## Results

### Subject characteristics and disposition

A total of 119 healthy male subjects were enrolled from September 19^th^, 2018, 117 subjects (98.3%) completed the study and lasted for follow-up until March 13^rd^, 2019.

The BSSR showed a coefficient of variation of 17.1%, which is less than the 25% considered for the sample size calculation so that the study could be completed with 40 patients per arm. Assuming that 95% of the subjects would be evaluable it was decided to enroll 126 subjects instead of 156. One subject in the US-sourced bevacizumab group was discontinued on Day 77 and another subject in the EU-sourced bevacizumab group on Day 88. All subjects were included (Fig 1) in the safety, immunogenicity, and PK analyses. The baseline demographic characteristics were similar among the three groups (Table 1).

**Table 1:** Demographic data (SA population).

		HD204	US-Avastin	EU-Avastin	Overall
		N = 40	N = 40	N = 39	N = 119
**Ethnicity n(%)**					
	Hispanic or Latino	3 (7.5%)	2 (5.1%)	2 (5.1%)	7 (5.9%)
	Not Hispanic or Latino	37 (92.5%)	37 (94.9%)	37 (94.9%)	111 (94.1%)
	Unknown	0	1	0	1
**Race n(%)**					
	Asian	9 (22.5%)	8 (20.0%)	4 (10.3%)	21 (17.6%)
	Black or African American	1 (2.5%)	0 (0.0%)	0 (0.0%)	1 (0.8%)
	Native Hawaiian or other Pacific	2 (5.0%)	0 (0.0%)	2 (5.1%)	4 (3.4%)
	White	27 (67.5%)	30 (75.0%)	26 (66.7%)	83 (69.7%)
	Other	1 (2.5%)	2 (5.0%)	7 (17.9%)	10 (8.4%)
**Age (years)**					
	Mean	27.9	26.8	28.2	27.6
	SD	7.7	6.2	8.5	7.5
	Median	25.0	26.0	25.0	76.0
	Min–Max	19–48	18–42	19–50	18–50
**Weight (kg)**					
	Mean	76.38	76.83	79.31	77.49
	SD	11.37	9.48	8.88	9.97
	Median	74.10	75.55	81.50	76.90
	Min–Max	60.3–99.9	62.1–98.1	60.9–99.7	60.3–99.9
**Height (cm)**					
	Mean	176.01	178.10	176.74	176.95
	SD	7.67	8.02	5.94	7.27
	Median	176.00	178.95	178.00	177.30
	Min–Max	159.0–192.7	159.0–195.0	165.1–188.4	159.0–195.0
**BMI (kg/m** ^**2**^ **)**					
	Mean	24.63	24.25	25.39	24.75
	SD	3.00	2.80	2.55	2.81
	Median	24.55	24.26	25.40	24.80
	Min–Max	18.9–30.0	19.3–29.6	20.7–29.8	18.9–30.0

BMI = body mass index; SD = standard deviation; Min = minimum; Max = maximum; N = number of subjects in the population.

For one subject in the EU-sourced bevacizumab group, an atypical PK concentration curve was observed with concentrations decreasing below the limit of quantification on Day 3, increasing after Day 4 to concentrations above the limit of quantification (350 ug/L), and reaching a maximum value of 65,900 ug/L on Day 22. It was confirmed by the laboratory that the samples were accurately labelled, and no documented reason could be identified for this atypical curve. No re-analysis of the samples was performed. This case was removed from the PK analysis. For personal reasons, sampling for one of the subjects in the HD204 group was only performed on Day 29, followed by an end of the study sample taken on Day 120, which showed a bevacizumab concentration below the limit of quantification. For this particular subject, since the sampling did not include Day 43, the AUC_0-last_ and all PK parameters were listed, but they were excluded from summaries/inferential analysis.

**Fig 2:**
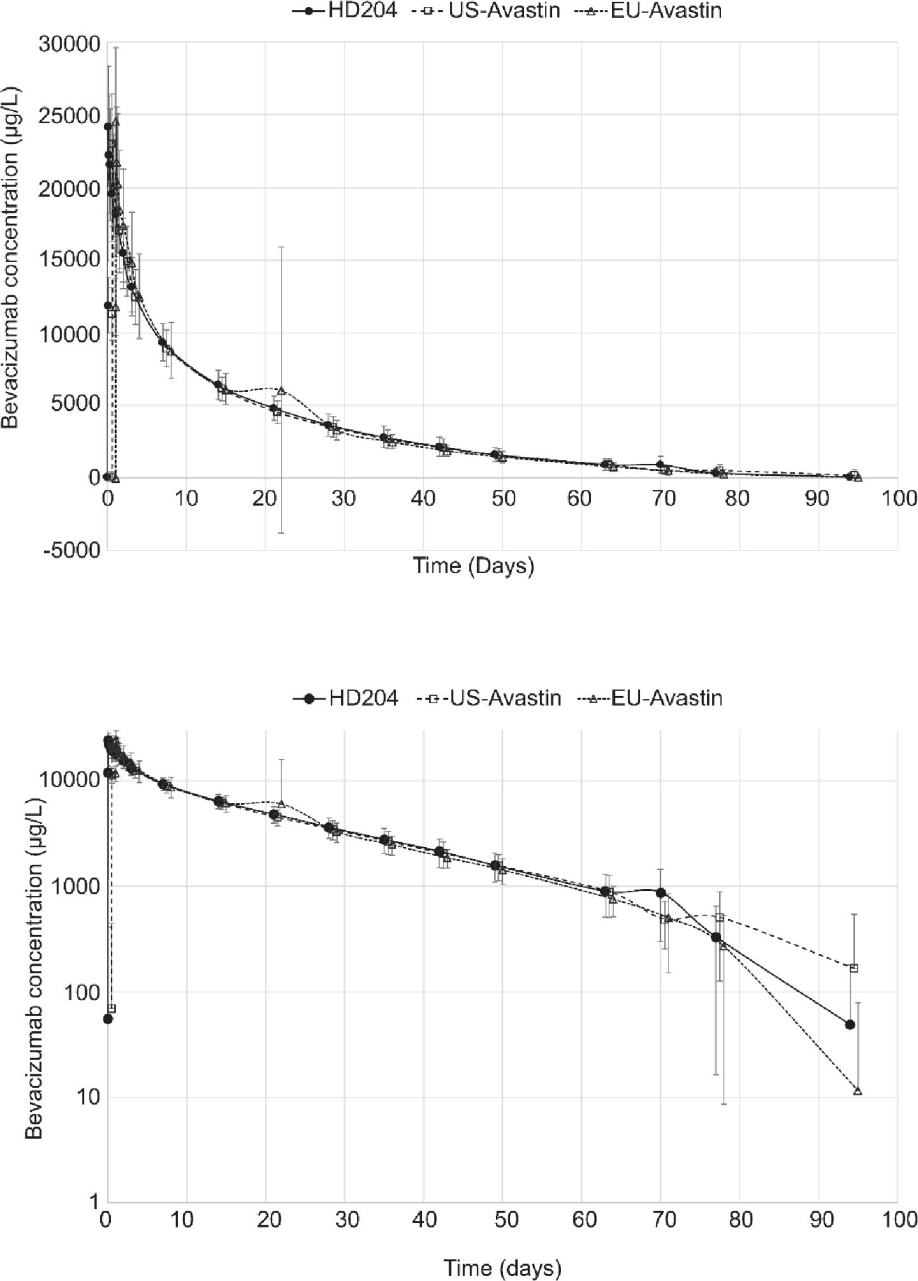
A-B Mean ± SD bevacizumab serum concentration-time profiles. Mean (±SD) bevacizumab concentrations over time are shown for all three groups on a linear scale (upper panel) and a semi-logarithmic scale (lower panel). Time 0h = pre-dose; Time 1.5h = end of infusion. Number of subjects with serum concentrations reported at each time are provided in the source table. Notes: Mean bevacizumab concentrations = 0.0 μg/L are not plotted on the semi-logarithmic graph; the large SD on Day 22 for EU-Avastin is due to one subject with an atypical PK profile; some error bars are not shown on the semi-logarithmic graph as negative values cannot be plotted logarithmically.

The mean (±SD) bevacizumab serum concentration-time profiles by treatment group are presented in Fig 2A and 2B.

Mean (±SD) of bevacizumab concentrations over time are shown for all three groups on a linear scale (upper panel) and a semi-logarithmic scale (lower panel). Time 0h = pre-dose; Time 1.5h = end of infusion. The number of subjects with serum concentrations reported at each time points is provided in the source table.

Notes: Mean bevacizumab concentrations = 0.0 μg/L are not plotted on the semi-logarithmic graph; the large SD on Day 22 for EU-Avastin is due to one subject with an atypical PK profile; some error bars are not shown on the semi-logarithmic graph as negative values cannot be plotted logarithmically.

Summary serum PK parameters by treatment groups are presented in Table 2.

**Table 2:** Summary of PK parameters for bevacizumab (PK population).

Parameter (units)		HD204 N = 39, n = 38	US-Avastin N = 39, n = 39	EU-Avastin N = 39, n = 39
AUC_0-inf_ (μg*h/mL)				
	Mean	7302.8	7083.6	7062.4
	SD	1213.6	1164.8	1467.2
	CV (%)	16.6	16.4	20.8
AUC_0-last_ (μg*h/mL)				
	Mean	6969.9	6752.7	6766.8
	SD	1183.9	1158.6	1455.3
	CV (%)	17.0	17.2	21.5
C_max_ (μg/mL)				
	Mean	24.29	22.99	25.59
	SD	4.18	3.47	8.35
	CV (%)	17.2	15.1	32.6
t_max_ (h)				
	Median	1.500	1.500	1.500
	Min—Max	1.50–7.52	1.50–13.60	1.50–505.40
λ_z_ (1/h)				
	Mean	0.001796	0.001719	0.001817
	SD	0.000243	0.000304	0.000184
	CV (%)	13.5	17.7	10.1
t_½_ (h)				
	Mean	392.33	415.37	385.4
	SD	49.93	76.29	38.72
	CV (%)	12.7	18.4	10.0
CL (L/h)				
	Mean	0.01066	0.01107	0.01153
	SD	0.00200	0.00177	0.00205
	CV (%)	18.7	16.0	17.8
V_ss_ (L)				
	Mean	5.57	6.058	5.857
	SD	1.040	1.315	0.886
	CV (%)	18.7	21.7	15.1
V_z_ (L)				
	Mean	6.002	6.600	6.378
	SD	1.096	1.653	1.096
	CV (%)	18.3	25.0	17.2

AUC_0-inf_ = area under the concentration-time curve from time 0 extrapolated to infinity; AUC_0-last_ = area under the concentration-time curve from time 0 to the last quantifiable data point; C_max_ = maximum observed concentration; t_max_ = time to maximum observed concentration; t_½_ = terminal half-life; λ_z_ = terminal rate constant.

All log-transformed variables hold for normality. For all other subjects, after administration of HD204, US-sourced, or EU-sourced bevacizumab, the percentage of the AUC_0-inf_ due to extrapolation was less than 20% of the overall AUC_0-inf_, demonstrating that the applied sampling schedule ensured that the majority of the AUC was captured and the range of times across which (_z_ was estimated was greater than twice the resultant t_½_. All summarized PK parameters were therefore considered to be reliably estimated.

AUC0-inf = area under the concentration-time curve from time 0 extrapolated to infinity; AUC0-last = area under the concentration-time curve from time 0 to the last quantifiable data point; Cmax = maximum observed concentration; tmax = time to maximum observed concentration; t½ = terminal half-life; λz = terminal rate constant;

The primary objective established equivalence in terms of pairwise comparisons of the ratios of geometric means between HD204 versus EU-sourced, HD204 versus US-sourced, and EU-sourced versus US-sourced bevacizumab. The mean values between the 3 arms differed by 3% (Table 3).

**Table 3:** Statistical analysis of PK properties of HD204, US-Avastin, and EU-Avastin (PKP population).

	Geometric LS Mean [95% CI]	Ratio (%) [90% CI]
**AUC**_**0-inf**_ (h*mg/L)		
HD204 (n = 38)	7205.4 [6821.2; 7611.4]	
US-Avastin (n = 39)	6999.0 [6630.4; 7388.1]	
EU-Avastin (n = 39)	6933.5 [6568.3; 7318.9]	
Pairwise Comparisons		
HD204 /US-Avastin		102.95 [96.52; 109.80]
HD204 /EU-Avastin		103.92 [97.43; 110.84]
EU-Avastin /US-Avastin		99.06 [92.92; 105.61]
**AUC**_**0-last**_ (h*mg/L)		
HD204 (n = 38)	6874.1 [6497.6; 7272.4]	
US-Avastin (n = 39)	6665.1 [6304.7; 7046.2]	
EU-Avastin (n = 39)	6635.6 [6276.8; 7015.0]	
Pairwise Comparisons		
HD204 /US-Avastin		103.14 [96.52; 110.20]
HD204 /EU-Avastin		103.59 [96.95; 110.69]
EU-Avastin /US-Avastin		99.06 [93.22; 106.33]
**C**_**max**_ **(ng/L)**		
HD204 (n = 39)	23.93 [22.46; 25.50]	
US-Avastin (n = 39)	22.75 [21.35; 24.24]	
EU-Avastin (n = 39)	24.65 [23.13; 26.27]	
Pairwise Comparisons		
HD204 /US-Avastin		105.57 [97.57; 113.41]
HD204 /EU-Avastin		97.08 [90.05; 104.67]
EU-Avastin /US-Avastin		108.36 [100.51; 116.82]

AUC_0-inf_ = area under the concentration-time curve from 0 to infinity; AUC_0-last_ = area under the concentration-time curve from 0 to last quantifiable analyte concentration; C_max_ = maximum plasma concentration immediately prior to the end of the infusion; LS Mean = least squares mean; CI = confidence interval, n = Number of subjects with the PK parameter.

The individual data point for each subject (AUC0–∞ AUc0–last C_max_) are shown in supplementary Fig 3A–3C. The inter-subject variabilities based on AUC_0-inf_ were moderate, with a coefficient of variation of approximately 16%. The unadjusted geometric LS mean AUC0–∞ values were 7205.4 mg*h/L (95% CI 6821.2; 7611.4), 6999 mg*h/L (95% CI 6630.4; 7388.1), and 6933.5 mg*h/L (95% CI 6568.3; 7318.9) for HD204, EU-sourced, and US-sourced Avastin, respectively. The ratio of the geometric mean of AUC_0-inf_ for HD204 versus EU-sourced bevacizumab was 103.92, with 90% CI [97.43% to 110.84%]. The ratio of the geometric mean of the primary PK parameter AUC_0-inf_ for HD204 versus US-sourced bevacizumab was 102.95, with 90% CI [96.52% to 109.80%]. Since the 90% CI of the ratio of geometric means of AUC_0-inf_ is contained within the acceptance range [80% to 125%], the equivalence of HD204 with EU-sourced and US-sourced bevacizumab can be concluded.

**Fig 3:**
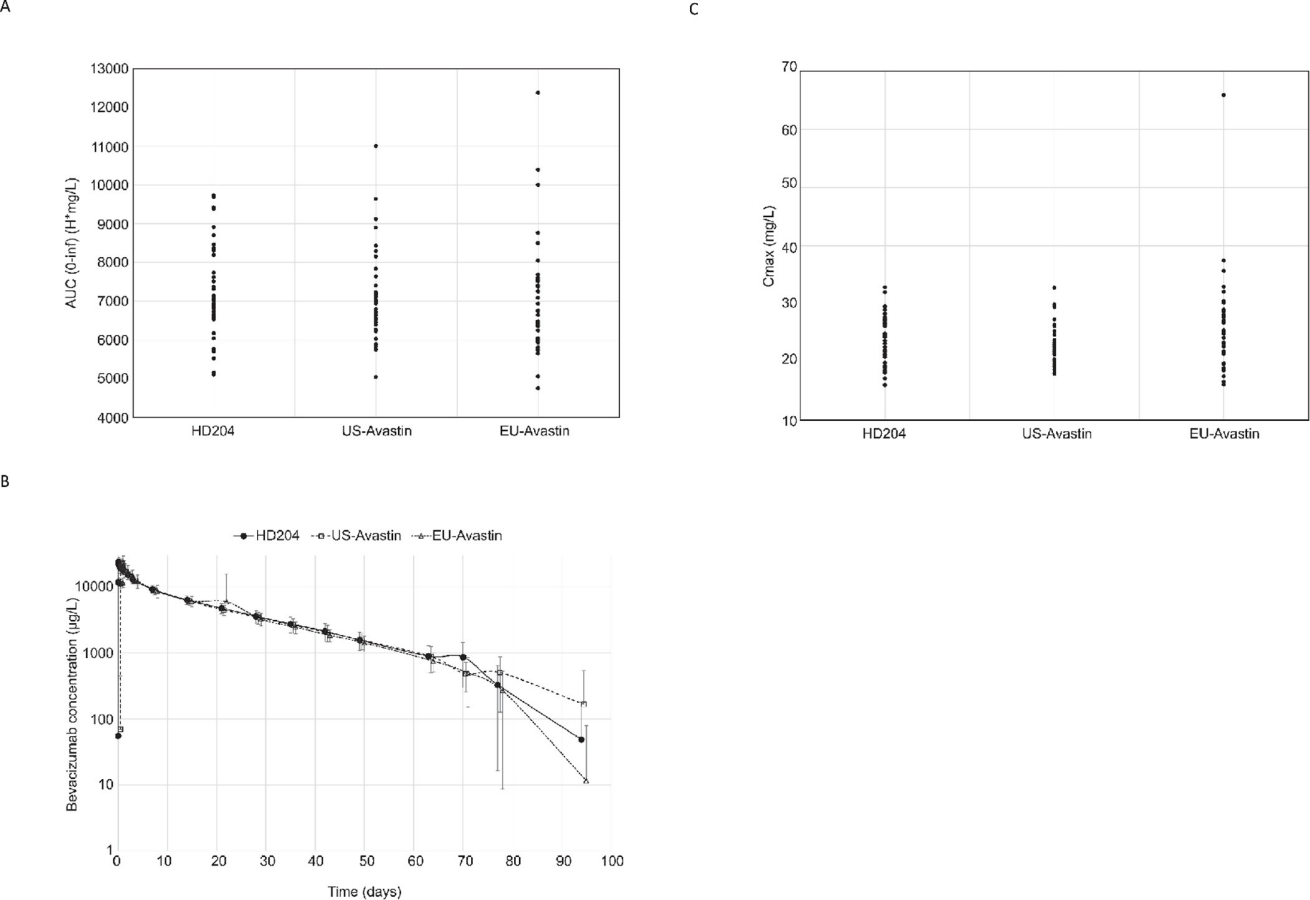


The ratio of the geometric mean of AUC_0-inf_ for EU-sourced versus US-sourced bevacizumab was 99.06, with 90% CI [92.92% to 105.61%].

Additionally, the 90% CI of the ratio of geometric means for the secondary PK parameters, AUC_0-last_ and C_max_, of HD204 versus US-Avastin, HD204 versus EU-Avastin, and EU-Avastin versus US-Avastin were all contained within the acceptance interval [80.00%; 125.00%] (Table 3). Therefore, based upon the secondary PK parameters, the pairwise equivalence of HD204, EU-sourced, and US-sourced bevacizumab can also be concluded.

### Safety

Adverse events (AEs) regardless of causality were reported in 31 (77.5%) subjects of the HD204 group, 31 (77.5%) subjects of the US-sourced bevacizumab group, and 34 (87.2%) subjects of the EU-sourced bevacizumab group (Table 4).

**Table 4:** Subjects with TEAEs by SOC and PT, reported ≥5% in any group.

		HD204		US-Avastin		EU-Avastin	
System Organ Class		N = 40		N = 40		N = 39	
Preferred Term		**n**	**%**	**n**	**%**	**n**	**%**
**Any TEAE**		**31**	**77.5%**	**31**	**77.5%**	**34**	**87.2%**
**Injury, poisoning, and procedural complications**		**18**	**45.0%**	**13**	**32.5%**	**13**	**33.3%**
	Contusion	4	10.0%	5	12.5%	4	10.3%
	Sunburn	5	12.5%	0	0.0%	1	2.6%
	Laceration	4	10.0%	0	0.0%	1	2.6%
	Skin abrasion	2	5.0%	2	5.0%	1	2.6%
	Arthropod bite	2	5.0%	1	2.5%	1	2.6%
	Muscle strain	0	0.0%	1	2.5%	3	7.7%
	Thermal burn	2	5.0%	2	5.0%	0	0.0%
**Infections and infestations**		**17**	**42.5%**	**8**	**20.0%**	**12**	**30.8%**
	Upper respiratory tract infection	12	30.0%	5	12.5%	8	20.5%
	Pharyngitis	0	0.0%	2	5.0%	0	0.0%
**Nervous system disorders**		**13**	**32.5%**	**9**	**22.5%**	**10**	**25.6%**
	Headache	9	22.5%	7	17.5%	7	17.9%
	Dizziness	4	10.0%	3	7.5%	1	2.6%
	Sensory disturbance	0	0.0%	0	0.0%	2	5.1%
**Musculoskeletal and connective tissue disorders**		**11**	**27.5%**	**5**	**12.5%**	**7**	**17.9%**
	Myalgia	3	7.5%	2	5.0%	1	2.6%
	Back pain	1	2.5%	2	5.0%	2	5.1%
	Arthralgia	3	7.5%	1	2.5%	0	0.0%
	Musculoskeletal pain	1	2.5%	0	0.0%	2	5.1%
**Skin and subcutaneous tissue disorders**		**6**	**15.0%**	**12**	**30.0%**	**4**	**10.3%**
	Dermatitis contact	2	5.0%	6	15.0%	1	2.6%
	Rash	0	0.0%	2	5.0%	1	2.6%
	Blister	0	0.0%	0	0.0%	2	5.1%
	Dermal cyst	0	0.0%	2	5.0%	0	0.0%
**General disorders and administration site conditions**		**10**	**25.0%**	**4**	**10.0%**	**6**	**15.4%**
	Vessel puncture site bruise	3	7.5%	2	5.0%	4	10.3%
	Fatigue	2	5.0%	1	2.5%	0	0.0%
**Respiratory, thoracic, and mediastinal disorders**		**9**	**22.5%**	**6**	**15.0%**	**5**	**12.8%**
	Nasal congestion	3	7.5%	2	5.0%	1	2.6%
	Oropharyngeal pain	2	5.0%	2	5.0%	2	5.1%
	Epistaxis	2	5.0%	1	2.5%	0	0.0%
**Gastrointestinal disorders**		**5**	**12.5%**	**7**	**17.5%**	**6**	**15.4%**
	Nausea	0	0.0%	2	5.0%	2	5.1%
	Diarrhoea	1	2.5%	0	0.0%	2	5.1%
	Toothache	0	0.0%	0	0.0%	2	5.1%
**Ear and labyrinth disorders**		**2**	**5.0%**	**0**	**0.0%**	**1**	**2.6%**
	Ear discomfort	2	5.0%	0	0.0%	0	0.0%

TEAE = treatment emergent adverse event; SOC = system organ class; PT = preferred term; N = number of subjects in the population; n = number of subjects with event.

Treatment-related AEs (TEAEs) were reported for 10 (25.0%), 12 (30.0%), and 10 (25.6%) subjects in the HD204, US-sourced and EU-sourced bevacizumab groups, respectively. There were no treatment-emergent serious AEs reported and no TEAEs leading to study discontinuation. Five subjects were reported to exhibit moderate TEAEs: 3 cases of infections in each arm; and 2 subjects with ligament sprain and muscle strain in the EU-sourced bevacizumab group.

#### Immunogenicity

None of the subjects in the HD204 group had positive ADA results. One subject each in the US-sourced and EU-sourced bevacizumab groups experienced positive ADA results. One subject tested positive for ADAs at all visits, 1 subject tested positive at all visits except on Day 64, and 1 subject tested positive for ADAs at pre-dose on Day 1 and then appeared negative on all subsequent visits. No neutralizing ADAs were found.

## Discussion

In this randomized, double-blind, parallel group, single dose study in healthy male subjects, PK equivalence between HD204 and the US-sourced and EU-sourced reference bevacizumab products was established. The study design, the findings, the limitations are comparable with the previously reported studies comparing a biosimilar bevacizumab candidate to its RMP. Generally, the problematic was comparable in PK equivalence studies assessing a biosimilar with a therapeutic antibody such not only bevacizumab but also trastuzumab or rituximab and the discussion developed below might overlap previously published manuscripts [6–10].

Addressing approximately 400 hours of t_1/2_ with bevacizumab, a parallel study design was preferred to save time, even if a cross-over design might have reduced the variability [7,11]. The majority of trials assessing the PK of biosimilar bevacizumab candidates adopted similar designs as well as trials evaluating other biosimilars of therapeutic antibodies such trastuzumab or rituximab [7–9,12–19]. In the present study, the inter-individual variability observed for the primary endpoint AUC_0-inf_ remained acceptable under the range of 20% of previously reported values. The study design has been considered appropriate for this type of research.

t is established that the quality of the RMP might be slightly variable over time, and differences might be observed between the EU-sourced and US-sourced RMP. These differences support the assessment of biosimilar candidates with a design including 3 arms. The 3-arms included the biosimilar candidate with both EU-sourced and US-sourced bevacizumab [12–14,16,17]. Similarly, to the present study, a PK equivalence was always demonstrated between EU-sourced and US-sourced bevacizumab in the planned pairwise comparison exercises. A multiplicity statistical correction should be considered to control the chance of making a type-1 error in a multiple arms study. Several conflicting viewpoints are expressed in the literature regarding the circumstances in which a multiple-testing correction should be used. In the present case, the study will conclude equivalence if only the 3 pairwise comparisons demonstrated equivalence. In this condition it was considered that no correction for multiplicity should be required.

Addressing AUC_0-inf_, the percentage due to extrapolation in the present study was inferior to 20% of the overall AUC_0-inf_. This limited extrapolated proportion supported the validity of the primary studied endpoint. In the present study, 90% CIs for the ratio of the geometric least square means of AUC_0-inf_ were included within the boundaries 80–125% and demonstrated equivalence. This interval of 80–125% for the LSMeans ratio to conclude PK equivalence were standard margins recommended by the regulatory authorities. Similarly, the 90% CIs for the ratio of the geometric least square means of AUC_0-last_ and C_max_ were contained within the same interval and supported the overall robustness of the PK equivalence. All other PK parameters were also comparable between HD204 and EU-sourced bevacizumab or US-sourced bevacizumab. The log-transformation data is widely used in biomedical research to deal with skewed data. In the present analysis the distribution of absolute differences between each AUC values as well as C_max_ values in all 3 arms hold normality. With these conditions, we might consider that the log transformed data should follow a normal distribution. Because using the log transformation reduces the variability, the distributions are nearly proportional pointing out the need to applied with cautious the data transformations to avoid violation of normal assumption for ANOVA test.

HD204 was well tolerated without serious AEs related to the study drug. The proportion of subjects who experienced AEs was also comparable between the 3 groups. Immunogenicity was assessed after study drug infusion, and no subject displayed a positive result for ADA. The overall low incidence of ADA is consistent with other study in healthy subjects assessing an unique infusion.

Overall, HD204 demonstrated an equivalent PK profile with both US-sourced bevacizumab and EU-sourced bevacizumab in healthy subjects. The next step requested by regulatory agencies in the development of this bevacizumab biosimilar candidate will be to establish an equivalence activity in a sensitive population [4,5]. Currently, metastatic non small cell lung cancer (NSCLC) was selected, and the randomized clinical trial SAMSON-II comparing paclitaxel–carboplatin with HD204 versus paclitaxel–carboplatin EU-sourced bevacizumab is ongoing (NCT03390686). Because several factors might theoretically impact the PK results, including administered dosages, regimen scheme (multiple *versus* single), chemotherapy partners, cancer setting and extension, a limited PK assessment in a subset of patients receiving iterative administration of bevacizumab or its biosimilar candidate in this large randomized study has been planned.

## Supporting information

S1 File Protocole HD204. (DOCX)
