# Supplementary material for: A randomized phase I study comparing the pharmacokinetics of a bevacizumab (HD204) biosimilar to European Union- and United States of America-sourced bevacizumab
Source: PLoS One. 2021 Sep 23;16(9):e0248222. doi: 10.1371/journal.pone.0248222 (PMC8460034; doi:10.1371/journal.pone.0248222)
Supplement: S1 File — (DOCX) [file pone.0248222.s001.docx]

**SYNOPSIS**

| Name of Sponsor: Prestige Biopharma | |  | | | |
| --- | --- | --- | --- | --- | --- |
| Name of Finished Product: HD204 | |  | | | |
| Name of Active Ingredient: Bevacizumab | |  | | | |
| TITLE OF STUDY | A Phase I, Double-blind, Randomized, Parallel Group Study to Demonstrate the Equivalent Pharmacokinetic Properties of a Single Intravenous Dose of HD204, US-Avastin® and EU-Avastin® in Healthy Male Subjects | | | | |
| PRINCIPAL INVESTIGATORS | Dr. Christian Schwabe and Dr. Chris Wynne | | | | |
| STUDY CENTERS | Auckland Clinical Studies  3 Fencroft St, Grafton, Auckland 1010, New Zealand  Christchurch Clinical Studies Trust Ltd  Level 4/264 Antigua Street, Christchurch 8011, New Zealand | | | | |
| STUDY PERIOD |  |  | | | |
| Clinical Phase Start: | 19 September 2018 | PHASE OF DEVELOPMENT: 1 | | | |
| Clinical Phase End: | 13 March 2019 | STUDY STATUS: Completed | | | |
| OBJECTIVES |  | | | | |
| Primary | The primary objective of the study was to establish pairwise pharmacokinetic (PK) similarity between HD204 (Prestige Biopharma Pte. Ltd bevacizumab), EU-Avastin, and US-Avastin after a single intravenous (IV) infusion of 1 mg/kg in healthy male subjects. | | | | |
| Secondary | The secondary objectives were to evaluate the safety, tolerability, and immunogenicity, and to further characterize the PK of HD204, EU-Avastin, and US-Avastin after a single IV infusion of 1 mg/kg in healthy male subjects. | | | | |
| METHODOLOGY | This was a randomized, double-blind, single dose, 3-way parallel group study in healthy male subjects aged 18 to 50 years, inclusive.  Each subject was randomly assigned to 1 of 3 treatment groups in a 1:1:1 ratio to receive a single IV infusion of 1 mg/kg of either HD204, EU-Avastin, or US-Avastin.  Subjects who had signed the informed consent form (ICF) and met all the eligibility criteria were admitted to the study center on Day -1 (i.e. the day prior to dosing). They received a single IV dose on Day 1 and were to be discharged 48 hours post-dose on Day 3. Thereafter, subjects were to attend the study center on an outpatient basis on Day 4, Day 8, Day 15, Day 22, Day 29, Day 36, Day 43, Day 50, Day 64, and Day 71.  Safety assessments included the evaluation of adverse events (AEs), vital signs, electrocardiogram (ECG) parameters, clinical safety laboratory tests (hematology, clinical biochemistry, coagulation, urinalysis [including proteinuria], and urine microscopy where clinically indicated), physical examination, injection site reactions, and concomitant medication. Blood samples were collected for PK evaluations, and measurement of antidrug antibodies (ADA) and neutralizing antibodies (NAb). | | | | |
| SAMPLE SIZE CALCULATION | Based on historical data, an inter-subject variability of 25% and a proportion of 20% of non-PK evaluable subjects were assumed for sample size determination. Assuming an inter-subject geometric coefficient of variation (CV) of 25% and a geometric mean ratio of 1.05, 40 evaluable subjects per arm were required for the 2-sided 90% confidence interval (CI) of the geometric mean ratio to be completely contained within 80.00% to 125.00% with 80% overall power and at least 90% power for each pairwise comparison (HD204 versus EU‑Avastin, HD204 versus US‑Avastin, and EU‑Avastin versus US‑Avastin). To allow for a proportion of 20% non-PK evaluable subjects, 150 subjects (50 subjects per arm) were planned to be enrolled.  Due to the uncertainty in the variability of the primary PK variable a blinded sample size re-evaluation (BSSR) was to be performed after 120 evaluable subjects (approximately 40 per arm) completed the study. If the BSSR showed that the total variability was at most equal to the planned variability of 25% CV considered for the sample size calculation the study was to be stopped. Otherwise, additional subjects were to be recruited.  The BSSR showed a CV of 17.1% which is less than the 25% considered for the sample size calculation so that the study could be completed as planned targeting 40 evaluable subjects per arm. Assuming that 95% of the subjects would be evaluable it was decided to enroll 126 subjects instead of 156. | | | | |
| RANDOMISATION | Each subject was randomly assigned to 1 of 3 treatment groups in a 1:1:1 ratio | | | | |
| NUMBER OF SUBJECTS | Planned for inclusion: 120 evaluable subjects were to be randomized, 40 in each treatment group  Screened and Randomized: 169 Consented; 119 Randomized. | | | | |
|  |  |  |  |  |  |
|  |  | **HD204** | **US-Avastin** | **EU-Avastin** | **Overall** |
|  | Consented Set |  |  |  | 169 |
|  | Safety Analysis (SA) Set | 40 | 40 | 39 | 119 |
|  | Pharmacokinetic Concentration (PKC) Set | 40 | 40 | 39 | 119 |
|  | Pharmacokinetic Parameter (PKP) Set | 39 | 39 | 39 | 117 |
|  |  |  |  |  |  |
| DIAGNOSIS & MAIN CRITERIA FOR INCLUSION & EXCLUSION | Inclusion Criteria  Subjects had to meet all the following criteria to be eligible for the study:   1. Adult healthy male aged 18 to 50 years (inclusive) and body mass index 18.0 to 30.0 kg/m^2^ (inclusive) and body weight ≥ 60 kg to ≤ 100 kg. 2. Is healthy as determined by pre-study medical history, physical examination, vital signs, and 12 lead ECG at screening and admission on Day 1. 3. Clinical laboratory test results are normal, or, where outside the reference range, are judged as not clinically relevant by the Investigator. 4. Have systolic blood pressure ≥ 90 and ≤ 140 mmHg, diastolic blood pressure ≥ 50 and ≤ 90 mmHg and heart rate ≥ 40 and ≤ 90 bpm at screening and admission on Day 1. For single measurements in the 141 to 160 mmHg range (systolic) or in the 91 to 100 mmHg range (diastolic), a single repetition on the same day is allowed and, in this case, the mean of both measurements will guide eligibility. The mean of both the measurements should be ≤ 140 mmHg (systolic) and ≤ 90 mmHg (diastolic). 5. Have physical examination results without clinically relevant findings at screening and admission on Day 1. 6. Have 12-lead ECG results without clinically relevant findings at screening and admission on Day 1. 7. Is non-smoker and has not regularly used tobacco or nicotine containing products for at least 3 months prior to screening and have a < 10 pack year smoking history. 8. Is willing to use a medically acceptable method of contraception from the time of the administration of study treatment, throughout the study and for a period of 6 months after the administration of the study treatment. This requirement may be waived if the Principal Investigator or delegate is satisfied that the subject or subject’s female partner is sterile i.e., if the female has undergone a hysterectomy or tubal ligation at least 3 months prior to screening or is postmenopausal (defined as no menses for 12 months without an alternative medical cause) or if the subject has undergone a vasectomy at least 6 months prior to screening. 9. Must agree not to donate sperm for at least 6 months after the administration of study treatment. 10. Must be willing and able to comply with scheduled visits, laboratory tests, and other study procedures. 11. Must be able to provide informed consent which must be obtained prior to any study-related procedures. 12. Is willing to comply with the study restrictions and comply with the scheduled visits from screening and throughout the study. | | | | |
|  | Exclusion Criteria  Subjects meeting any of the following criteria were not eligible for the study:   1. Has a history of hypersensitivity or allergic reactions (either spontaneous or following drug administration) to bevacizumab or VEGF targeted treatment or to any other excipients. 2. Has a history of or presence of current clinically significant gastrointestinal (including diverticulitis, stomach ulcers, inflammatory intestinal disease, gastrointestinal perforations/fistulae/intra-abdominal abscess), any other internal, non-gastrointestinal fistulae that is at an increased risk of bleeding, renal, hepatic, cardiovascular, hematological (including pancytopenia, aplastic anemia or blood dyscrasia), pulmonary, neurologic, metabolic (including known diabetes mellitus), psychiatric or allergic disease excluding mild asymptomatic seasonal allergies. 3. Has a history of and/or current cardiac disease defined as one of the following:   • History of congestive heart failure  • Angina pectoris requiring antianginal medication  • Evidence of transmural infarction on ECG  • History of sustained hypertension (systolic > 180 mmHg and/or diastolic > 100 mmHg) or hypertensive crisis or hypertensive encephalopathy  • Clinically significant valvular heart disease  • Severe arterial thromboembolic events   1. Has a positive test result for hepatitis B surface antigen (HBsAg), hepatitis C virus, or human immunodeficiency virus (HIV) I and II at screening. 2. Has any history of malignancy including lymphoma, leukemia, and skin cancer (even resected basal cell carcinoma or squamous cell carcinoma). 3. Has an illness within 30 days prior to screening, or prior to Day -1, that is considered as clinically significant by the Investigator. 4. Prior exposure to any investigational monoclonal antibody within 12 months of Day -1. Prior investigational exposure to Avastin > 12 month is acceptable. 5. Any clinically significant infection, in the opinion of the Investigator, ongoing at screening or admission on Day -1. 6. Has had major surgery within 3 months prior to screening or will have an operation between screening and the end of study visit, or still has an unhealed wound, including wound dehiscence and wound healing complications requiring medical intervention. Major Surgery would include any surgery which is invasive, and allows access to body cavities beyond the skin, superficial tissues or mucous membranes, requires beyond local anesthesia, or requires in house hospitalization. 7. Have received live vaccine(s) within 3 months prior to screening or will require a live vaccine(s) between screening and the end of study visit. 8. Has an intake of alcoholic beverages of more than 14 units per week (1 unit = 250 mL of beer, 25 mL of spirits or 1 glass [125 mL] of wine). 9. Has reasonable evidence (in the opinion of the Investigator) of drug abuse as indicated by a positive urinary drug test at screening or admission on Day -1. 10. Has taken medication with a half-life of >24 hours within 30 days or less than 10 half-lives of the medication prior to Day -1 as determined by the Investigator. 11. Has donated blood and blood products within 3 months prior to Day -1. Blood donation and blood products are not permitted throughout study. 12. Has participated in another clinical study of an investigational drug within 90 days or 5 half-lives of the investigational drug (whichever is longer) prior to Day -1 or are currently participating in another clinical study of an investigational drug or intending to participate in another clinical study of an investigational drug before completion of all scheduled evaluations in this clinical study. 13. In the opinion of the Investigator, is not likely to complete the study for whatever reason. 14. Is the Investigator or any sub-investigator, research assistant, pharmacist, study coordinator, other staff directly involved in the conduct of the clinical study or any of their immediate family members (parents, siblings or spouses). 15. Abnormal or irregular bowel movements e.g., less than 1 bowel movement every 3 days. 16. Any history of non-traumatic hemorrhage (i.e., any hemorrhage requiring medical intervention) or any condition which may increase bleeding risk including clotting disorders, thrombocytopenia (platelet count < 150, 000 per µL) or an international normalized ratio higher than 1.5 at screening. 17. Impaired liver function as determined by: Serum ALT and/or AST > 1.5 × ULN at screening or admission. Subjects with values between ULN and 1.5 × ULN may be included in the study if considered not clinically significant by the Investigator. 18. Any intake of a non-steroidal anti-inflammatory drug (NSAID) including any dose of aspirin in the 30 days before Day -1 (NSAIDs are not allowed for the duration of the study). 19. Intake of herbal drugs or dietary supplements excluding routine vitamins but including megadose (intake of 20 to 600 times the recommended daily dose) vitamin therapy within 30 days prior to Day -1, unless agreed as not clinically relevant by the Investigator and Sponsor. 20. Strenuous exercise or activity within 96 hours prior to admission on Day -1 and has a creatinine kinase value > 2.5 × ULN. 21. Has unsuitable veins for infusion or venipuncture. 22. Has a non-healing wound or hematoma of a clinically relevant size as assessed by the Investigator, or a clinically significant current fracture. 23. Presence of proteinuria (other than trace amounts i.e., +, ++/+++) at screening or admission on Day -1. 24. Known personal or family history of venous thromboembolic events or idiopathic venous thromboembolic events in first degree relatives. 25. Inability to refrain from contact or collision sport until Day 30. | | | | |
| TEST PRODUCT | HD204 (bevacizumab) | | | | |
| REFERENCE PRODUCTS | EU-sourced Avastin (bevacizumab)  US-sourced Avastin (bevacizumab) | | | | |
| DOSE | For each product (test and references): sterile solution containing 400 mg/16 mL, of which a single dose of 1 mg/kg | | | | |
| MODE OF ADMINISTRATION | IV infusion | | | | |
| BATCH NUMBERS | HD204: HD204P-1801, expiry date: 25 April 2020  EU-Avastin: B8030H16, expiry date: April 2019  US-Avastin: 3228637, expiry date: January 2020 | | | | |
| DURATION OF TREATMENT | The treatment consisted of a single 90-minute infusion. The duration of participation for each enrolled subject was maximum 120 days, comprising a 28-day screening period, a single treatment day, and a 95-day follow-up period. | | | | |
| ENDPOINTS |  | | | | |
| Efficacy | Efficacy was not evaluated in this study. | | | | |
| Pharmacokinetics | The following PK parameters were estimated:   \| AUC_0-inf_ \| Area under the concentration-time curve in serum from zero (pre-dose) extrapolated to infinite time (µg·h/mL). \| \| --- \| --- \| \| AUC_0-last_ \| Area under the serum concentration-time curve from time zero to the time of last quantifiable analyte concentration (µg·h/mL) \| \| C_max_ \| Maximum concentration in serum (µg/mL) \| \| t_max_ \| Time of maximum concentration (h) \| \| λ_z_ \| Terminal rate constant (1/h) \| \| t_1/2_ \| Terminal half-life (h) \| \| CL \| Total systemic clearance (L/h) \| \| V_ss_ \| Apparent volume of distribution at steady state (L) \| \| V_z_ \| Volume of distribution following intravenous dosing (L) \|   AUC_0-inf_ is the primary PK parameter. | | | | |
| Safety | Safety and tolerability were assessed by:   - Clinical laboratory tests - Vital signs - 12‑lead ECGs - Physical examinations - Assessment of AEs - Injection site reactions - Use of concomitant medications. | | | | |
| STATISTICAL METHODS |  | | | | |
| Analysis sets | The **Safety Analysis (SA)** **Population** consists of all randomized subjects who received study treatment (partial or whole).  The **Pharmacokinetic Concentration (PKC)** **Population** includes subjects with at least 1 valid post-dose concentration, have received 1 dose of study drug without major protocol violations which may significantly affect the PK assessment. Subjects are excluded from the PKC population if they have a significant protocol deviation that is likely to impact concentration. Subjects confirmed positive post-dose for anti-bevacizumab antibodies were not excluded from the PKC population.  The **Pharmacokinetic Parameter (PKP) Population** is a subpopulation of the PKC population who have at least one evaluable primary or secondary PK parameter.  The SA population was used for the analysis of safety and immunogenicity.  Serum bevacizumab concentrations summaries were based on the PKC population. Pharmacokinetic parameter summary statistics and assessment of PK equivalence were based on the PKP population. The analysis was repeated for secondary parameters C_max_ and AUC_0-last_. | | | | |
| Onset data | Summary statistics are presented for demographic data (ethnicity, race, age, weight, height, and BMI). Medical History was summarized by MedDRA System Oran Class (SOC) and Preferred Term (PT). | | | | |
| Pharmacokinetics | A subject listing of all concentration-time data for each treatment is presented. Figures of arithmetic mean concentration-time data (±SD, as appropriate) are presented for each treatment on linear and semi‑logarithmic scales. Individual subject concentration-time data are represented graphically on linear and semi‑logarithmic scales.  Pharmacokinetic parameters are summarized based on the PKP population by treatment using descriptive statistics. A subject listing of individual PK parameters for each treatment is provided. Scatter plots of individual and geometric mean PK parameters (AUC_0-inf_, AUC_0-last_, and C_max_) versus treatment are presented.  The primary statistical null hypothesis is that AUC_0-inf_ is not equivalent between HD204 and the reference products within 80.00% to 125.00% limits, against the alternative that HD204 is equivalent to the reference products.  The primary PK parameter, AUC_0-inf_, is compared using an analysis of variance (ANOVA) model with treatment as a fixed effect. The data are natural log transformed prior to the analysis. Transformed back to the original scale, the geometric mean together with the 2-sided 95% CI for each treatment is estimated and presented. Also, ratios of geometric means together with CIs (2-sided 90%) for treatment comparisons (HD204 versus EU-Avastin, HD204 versus US-Avastin, EU-Avastin versus US‑Avastin) are estimated and presented.  The 90% CI of the ratio of geometric means of log-transformed AUC_0-inf_ is used to assess bioequivalence between the test and reference using the bioequivalence interval of 80.00% to 125.00%. Bioequivalence is declared if the 90% CI for the ratio falls within 80.00% and 125.00% for the primary PK parameter AUC_0-inf_ for the comparisons: HD204 versus EU-Avastin, HD204 versus US-Avastin. The analysis is repeated for secondary parameters C_max_ and AUC_0-last_.  For the BSSR, PK parameters are estimated using nominal (scheduled) times if actual times are not available at that stage. Protocol deviations, adverse events, concomitant medications plus other relevant study data available are reviewed to determine if subjects should be excluded from the analysis. Descriptive statistics are generated to summarize across treatment arms and estimate the overall geometric CV. | | | | |
| Safety | Adverse events  A summary is provided for the frequencies of subjects with any TEAE, any treatment-related TEAE, any treatment-emergent SAE, any treatment-emergent SAE by type of SAE, any treatment-related treatment-emergent SAE, any mild, moderate, and severe TEAE, and any TEAE leading to study discontinuation. Summaries are also provided of subjects with TEAEs by SOC and PT:   - Any TEAE - Any treatment-related TEAE - Any TEAE by severity - Any treatment-related TEAE by severity   Clinical safety laboratory data  Quantitative laboratory parameters are summarized descriptively by scheduled time-point and for the change from baseline. Frequencies of occurrences outside the normal range are summarized by scheduled time-point.  Vital signs, electrocardiogram, and physical examination  Vital signs (blood pressure, heart rate, respiratory rate, and temperature) are summarized descriptively by scheduled time-point and for the change from baseline.  Quantitative ECG assessments (heart rate, PR interval, QRS duration, QT interval, and QTcF interval) are summarized descriptively by scheduled time-point and for the change from baseline. The overall assessment (’Normal’, ’Abnormal, Not Clinically Significant’, or ’Abnormal, Clinically Significant’), is summarized by time-point.  Physical examination findings are listed by body system and time-point, including specification of any abnormalities observed. | | | | |
| Safety | TEAEs were reported in 31 (77.5%) subjects of the HD204 group, 31 (77.5%) subjects of the US-Avastin group, and 34 (87.2%) subjects of the EU-Avastin group.  Treatment related TEAEs were reported for 10 (25.0%) subjects of the HD204 group, 12 (30.0%) subjects of the US-Avastin group, and 10 (25.6%) subjects of the EU-Avastin group. There were no treatment-emergent SAEs reported and no TEAEs leading to study discontinuation. There were no severe TEAEs and all TEAEs related to study drug were of mild intensity. Only one subject treated with HD204 had an injection site reaction that was possibly related to study drug. The event was of mild severity and lasted from Day 1 till Day 3.  There were no deaths during the study.  There were no notable differences between the three treatment arms in vital signs, ECGs, or laboratory tests.  None of the subjects treated with HD204 had positive ADA results. | | | | |

**LIST OF ABBREVIATIONS AND DEFINITION OF TERMS**

| **ABBREVIATION** | **DEFINITION** |
| --- | --- |
| ADA | Anti-Drug Antibodies |
| ADCC | Antibody-Dependent Cell-mediated Cytotoxicity |
| AE | Adverse Event |
| ANOVA | ANalysis of VAriance |
| AUC | Area Under the Curve (plasma concentration-time profile) |
| AUC_0-t_ | Area Under the Curve (concentration-time curve from time 0 to last quantifiable concentration) |
| AUC_0-inf_ | Area Under the Curve (concentration-time curve from 0 to infinity) |
| A_z_ | Terminal phase rate constant |
| BLQ | Below Limit of Quantification |
| BMI | Body Mass Index |
| CI | Confidence Interval |
| CL | Total clearance |
| C_max_ | Maximum plasma concentration immediately prior to the end of the infusion |
| CRF | Case Report Form |
| CRO | Clinical Research Organisation |
| CV (%) | Coefficient of Variation, expressed as a percentage |
| ECG | ElectroCardioGram |
| ELISA | Enzyme-Linked Immunosorbent Assay |
| FITC | Fluorescein IsoThioCyanate |
| GCP | Good Clinical Practice |
| GMP | Good Manufacturing Practice |
| HBsAg | Hepatitis B surface Antigen |
| HER2 | Human Epidermal Growth Factor 2 |
| HIV | Human Immunodeficiency virus |
| ICF | Informed Consent Form |
| ICH | International Conference on Harmonisation |
| IV | Intravenous |
| MedDRA | Medical Dictionary for Regulatory Activities |
| NAb | Neutralising Antibodies |
| PD | Pharmacodynamics |
| PK | Pharmacokinetics |
| PKC | Pharmacokinetic Concentration population |
| PKP | Pharmacokinetic Parameter population |
| PKNN | Pharmacokinetic Non-Neutralizing population |
| PT | Preferred Term |
| QC | Quality Control |
| SA | Safety Analysis population |
| SAE | Serious Adverse Event |
| SD | Standard Deviation |
| SOC | System Organ Class |
| SOP | Standard Operating Procedure |
| TEAE | Treatment-Emergent Adverse Event |
| t_½_ | Half-life |
| UK | United Kingdom |
| US | United States |
| V_d_ | Volume of distribution |
| VEGF | Vascular Endothelial Growth Factor |
|  |  |
|  |  |
|  |  |

**ETHICS**

**Independent Ethics Committee**

The final study protocol dated 23 August 2018 (Version 2.0) and informed consent form (ICF) were reviewed and approved prior to study commencement by the Health and Disability Ethics Committees, Ministry of Health, Wellington, New Zeeland, on 27 August 2018.

The amended study protocol dated 23 January 2019 (Version 3.0) and informed consent form (ICF) were reviewed and approved by the Health and Disability Ethics Committees, Ministry of Health, Wellington, New Zeeland, on 18 February 2019.

The protocol was submitted for consideration by Medsafe, the New Zealand Medicines and Medical Devices Safety Authority, from which written approval was obtained on 17 August 2018.

**Ethical Conduct of the Study**

This study was conducted in accordance with the ethical principles that have their origin in the Declaration of Helsinki on biomedical research involving human volunteers (6th revision [2008]) and Council for International Organizations of Medical Sciences (CIOMS) International Ethical Guidelines and are consistent with the International Council for Harmonisation of Technical Requirements for Registration of Pharmaceuticals for Human Use (ICH) Good Clinical Practice (GCP) guidelines (CPMP/ICH/135/95), applicable local regulatory requirements and laws, and IQVIA standard operating procedures (SOPs).

**Participant Information and Informed Consent**

Written informed consent was obtained from each subject at the screening visit prior to the initiation of any study-related procedures:

- The Investigator or his/her representative explained the nature of the study to the subject and answered all questions regarding the study.
- Subjects were informed that their participation was voluntary. Subjects were required to sign a statement of informed consent that met the requirements of 21 CFR 50, local regulations, ICH guidelines, Health Insurance Portability and Accountability Act (HIPAA) requirements, where applicable, and the IRB/IEC or study center.
- In the medical record a statement was noted that written informed consent was obtained before the subject was entered in the study and the date the written consent was obtained. The authorized person obtaining the informed consent also signed the ICF.
- If applicable, subjects were to re-consent to the most current version of the ICF(s) during their participation in the study.
- A copy of the ICF(s) was provided to the subject.
